# Unlocking precision medicine: clinical applications of integrating health records, genetics, and immunology through artificial intelligence

**DOI:** 10.1186/s12929-024-01110-w

**Published:** 2025-02-07

**Authors:** Yi-Ming Chen, Tzu-Hung Hsiao, Ching-Heng Lin, Yang C. Fann

**Affiliations:** 1 Division of Allergy, Immunology and Rheumatology, Department of Internal Medicine, Taichung Veterans General Hospital, Taichung, 40705 Taiwan; 2 School of Medicine, National Yang Ming Chiao Tung University, Taipei, 11221 Taiwan; 3 Department of Medical Research, Taichung Veterans General Hospital, Taichung, 40705 Taiwan; 4 Department of Post-Baccalaureate Medicine, College of Medicine, National Chung Hsing University, Taipei, 112304 Taiwan; 5 Graduate Institute of Clinical Medicine, College of Medicine, National Chung Hsing University, Taichung, 402202 Taiwan; 6 Precision Medicine Research Center, College of Medicine, National Chung Hsing University, Taichung, 402202 Taiwan; 7 Department of Public Health, College of Medicine, Fu Jen Catholic University, New Taipei City, 242062 Taiwan; 8 Institute of Genomics and Bioinformatics, National Chung Hsing University, Taichung, 402202 Taiwan; 9 Department of Industrial Engineering and Enterprise Information, Tunghai University, Taichung, 407224 Taiwan; 10 Institute of Public Health and Community Medicine Research Center, National Yang Ming Chiao Tung University, Taipei, 11221 Taiwan; 11 Division of Intramural Research, National Institute of Neurological Disorders and Stroke, National Institutes of Health, Bethesda, MD 20892 USA

**Keywords:** Artificial intelligence, Precision medicine, Electronic health records, Genetics, Immunology, Autoimmune

## Abstract

Artificial intelligence (AI) has emerged as a transformative force in precision medicine, revolutionizing the integration and analysis of health records, genetics, and immunology data. This comprehensive review explores the clinical applications of AI-driven analytics in unlocking personalized insights for patients with autoimmune rheumatic diseases. Through the synergistic approach of integrating AI across diverse data sets, clinicians gain a holistic view of patient health and potential risks. Machine learning models excel at identifying high-risk patients, predicting disease activity, and optimizing therapeutic strategies based on clinical, genomic, and immunological profiles. Deep learning techniques have significantly advanced variant calling, pathogenicity prediction, splicing analysis, and MHC-peptide binding predictions in genetics. AI-enabled immunology data analysis, including dimensionality reduction, cell population identification, and sample classification, provides unprecedented insights into complex immune responses. The review highlights real-world examples of AI-driven precision medicine platforms and clinical decision support tools in rheumatology. Evaluation of outcomes demonstrates the clinical benefits and impact of these approaches in revolutionizing patient care. However, challenges such as data quality, privacy, and clinician trust must be navigated for successful implementation. The future of precision medicine lies in the continued research, development, and clinical integration of AI-driven strategies to unlock personalized patient care and drive innovation in rheumatology.

## Introduction

Precision medicine marks a pivotal shift in healthcare, steering away from the conventional ‘one-size-fits-all’ approach to a more personalized strategy tailored to individual patient profiles with immune disorders [1, 2]. This approach is deeply rooted in the integration of multi-dimensional data sources, including genetic information, immunological profiles, and extensive health records. The importance of integrating these diverse data types is magnified by the application of artificial intelligence (AI), which significantly enhances our ability to interpret complex biological data and translate it into clinically actionable insights [1].

AI, particularly through its subsets of machine learning (ML) and deep learning, is instrumental in dissecting the multi-omics and multifaceted datasets typical of modern healthcare systems [3]. These technologies excel at recognizing patterns and anomalies within large datasets, facilitating more precise and predictive healthcare. In genetics, AI algorithms are crucial for analyzing genetic markers quickly and accurately, offering insights into patient susceptibilities and potential responses to treatments [4]. This capability not only accelerates the process of genetic screening but also enhances the specificity of therapeutic interventions tailored to individual genetic profiles.

In the domain of immunology, AI’s impact is equally transformative. AI models are adept at simulating immune system dynamics and predicting how it might respond to various treatments. This is particularly valuable in the design of personalized immunotherapies, which aim to optimize efficacy while minimizing adverse effects, thereby significantly advancing patient care in conditions such as autoimmune diseases and cancer immunotherapies [3, 5–7].

Moreover, the integration of AI with electronic health records (EHRs) provides a comprehensive overview of patient health histories, enhancing diagnostic accuracy and treatment efficacy [8]. AI-driven analysis of EHRs can identify hidden patterns across patient populations, predict disease progression, and suggest preventative measures tailored to individual health profiles [9]. This holistic approach not only improves individual patient outcomes but also contributes to broader public health strategies.

However, while the integration of AI into precision medicine offers substantial benefits, it also brings challenges such as ensuring data privacy, securing patient consent, and managing the ethical complexities associated with AI-driven decisions. The success of AI in healthcare hinges on navigating these challenges carefully, maintaining the integrity of patient data, and ensuring that AI-driven interventions are transparent and equitable [10].

**Fig. 1:**
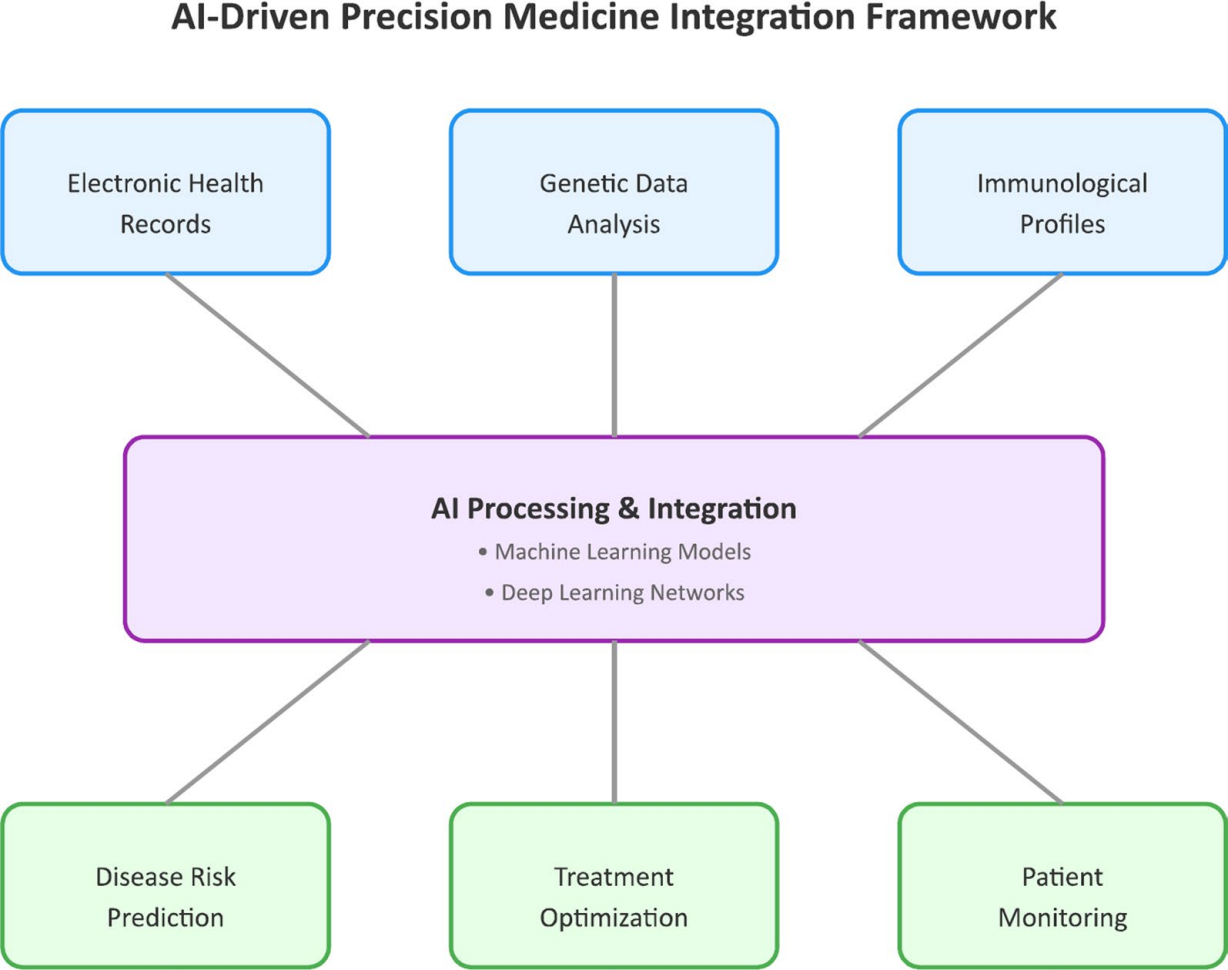
Framework for AI-Driven Integration of Multi-modal Data in Precision Medicine

The aim of this comprehensive review is to explore the clinical applications of integrating health records, genetics, and immunology in precision medicine, facilitated by the advancements in AI, for patients with autoimmune rheumatic diseases (Fig. 1). This review will examine how AI-driven analytics can transform the landscape of precision medicine by offering more accurate prognostics and targeted therapeutic interventions.

### AI-driven analysis of health records

AI has made significant strides in healthcare, offering innovative solutions to enhance patient care and streamline medical processes. We can see numerous successful applications of AI in healthcare, all built upon vast Electronic Health Records (EHR). Especially, deep learning has gained major attention due to its remarkable success in tasks like face recognition, image classification, object detection, and segmentation [11]. These advancements stem from deep learning models’ ability to automatically learn intricate patterns in extensive datasets, enabling highly accurate and complex visual recognition. This success has spurred AI adoption across diverse fields. For example, Aidoc’s AI platform transforms radiology departments by providing continuous, intelligent support, allowing radiologists to make faster and more accurate decisions. It analyzes CT scans to identify critical conditions like strokes, pulmonary embolisms, and brain hemorrhages, facilitating timely interventions [12]. Similarly, Google’s AI system has achieved a breakthrough in diabetic retinopathy screening, examining retinal photos with accuracy comparable to ophthalmologists, enabling early detection and treatment to potentially prevent vision loss [13]. Beyond image recognition, AI requires real EHR data to support its judgments and remove noise; without this, AI cannot learn effectively.

In addition to imaging applications, AI also shows promise in drug discovery and epidemiological forecasting. For example, Atomwise’s platform, AtomNet, uses deep learning to analyze molecular structures and predict effective molecules for drug development, potentially providing treatments for diseases like Ebola and multiple sclerosis [14]. BlueDot’s AI platform, meanwhile, monitors global health trends to predict the spread of infectious diseases, offering insights that support proactive healthcare measures [15]. These models rely on collecting various types of EHR data and repeated long-term measurements.

The success of AI in healthcare can be attributed to its ability to process vast amounts of data swiftly and accurately. Before modeling, the application of AI in EHR requires several steps (Table 1):

**Table 1:** The Steps for Applying AI to Process EHR

Step	Aim	Role of AI
Step 1: Data collection	Gather comprehensive data from multiple sources	ML algorithms extract information from unstructured EHR data. NLP models identify key terms
Step 2: Data cleaning	Address missing values, remove duplicates, and fix inconsistencies	AI corrects errors
Step 3: Normalization and standardization	Adjust data to a standard scale for comparability	AI models normalize and standardize quantitative data
Step 4: Data preservation	Maintain integrity and confidentiality of data	AI helps monitor and secure data throughout the process

#### Step 1: data collection

The process begins with data collection, where EHRs are aggregated from sources like hospitals, clinics, and healthcare providers. Leveraging AI, particularly ML, greatly enhances data collection from EHRs. ML algorithms can extract vital information from unstructured EHR data, such as clinical notes, lab reports, and radiology images. Natural Language Processing (NLP) models can identify key terms, diagnoses, medications, and patient demographics. This automation streamlines the data collection process and reduces the need for manual effort [16, 17].

#### Step 2: data cleaning

Following data collection, data cleaning is essential. This phase involves meticulously addressing missing values, eliminating duplicates, and rectifying any inconsistencies. AI can significantly enhance data cleaning in EHRs by correcting errors like misspelled units through fuzzy search and unit conversion, as well as using clinical context to clean numeric values and detect outliers. The goal is to achieve high automation while maintaining data quality and ensuring alignment with data governance policies [18].

#### Step 3: normalization and standardization

Normalization and standardization are subsequent steps that adjust numerical data to a common scale. This is especially important for lab results and other quantitative measures, ensuring that all data points are comparable and interpretable by AI models [19].

#### Step 4: data preservation

Maintaining the integrity and confidentiality of patient data is essential throughout this process.

Each of these steps plays a vital role in preparing data for AI processing. Together, they ensure that the data fed into AI algorithms is high-quality, structured appropriately, and contains the most relevant information for analysis.

### Potential liability and reimbursement

The implementation of AI in EHR systems raises critical considerations regarding liability and reimbursement. From a liability perspective, the use of AI-driven clinical decision support tools creates complex questions about responsibility when adverse events occur. Healthcare providers must navigate situations where AI recommendations conflict with clinical judgment, requiring clear protocols for documentation and decision-making processes. Medical institutions need robust frameworks to determine liability distribution among healthcare providers, AI system developers, and healthcare organizations. This includes establishing clear audit trails for AI-assisted decisions and maintaining transparency in algorithmic decision-making processes. Regarding reimbursement, the integration of AI tools into clinical practice faces several challenges. Current healthcare payment systems are not fully equipped to handle AI-assisted services, with limited Current Procedural Terminology (CPT) codes specifically designed for AI applications. While some insurers have begun covering AI-assisted diagnostics, particularly in medical imaging, reimbursement policies for AI applications in clinical decision support remain inconsistent. Healthcare organizations must carefully evaluate the cost-effectiveness of AI implementations, considering both the initial investment and ongoing operational costs against potential reimbursement rates. Recent studies suggest that value-based care models may better accommodate AI-enabled services compared to traditional fee-for-service systems. The Centers for Medicare and Medicaid Services (CMS) has started developing frameworks for AI reimbursement, but standardization across different payers remains a significant challenge. These considerations underscore the need for healthcare organizations to develop comprehensive strategies addressing both liability protection and financial sustainability when implementing AI-driven EHR systems.

### AI-powered genetics analysis

Genetics have undergone a revolutionary transformation in recent years, driven by rapid advancements in high-throughput sequencing technologies and the exponential growth of genomic data [20]. As those national-wide database, such as UK Biobank and All of US, demonstrated, the vast amount of information generated by these techniques has created an unprecedented opportunity for researchers to unravel the intricate relationships between genetic variations phenotypic traits and diseases, paving the way for personalized medicine and precision healthcare [21, 22]. However, the sheer volume and complexity of genomic data have also posed significant challenges in data analysis and interpretation [23, 24].

AI, a powerful computational paradigm that has shown remarkable prowess in extracting insights from large and complex datasets [25, 26]. AI techniques, such as machine learning and deep learning, have recently emerged as invaluable tools for navigating the genomic landscape, enabling researchers to identify patterns, make predictions, and uncover hidden relationships within the vast expanse of genetic information [4]. For instance, AI can help in analyzing genomic sequences to predict the impact of genetic variations on protein structure and pathogenicity, and pinpointing potential active pathway. Additionally, AI-driven tools are improving the efficiency and accuracy of genome techniques such as CRISPR and neoantigen identification, accelerating the pace of genetic research and the development of novel treatments for genetic disorders. In this review, we provide a comprehensive overview of the current state-of-the-art AI methodologies in variant calling and MHC-Peptide binding, highlighting the potential benefits of these approaches (Table 2).

**Table 2:** Summary of AI-powered Genetics Analysis

Section	Description	Key AI Tools	Applications
Variant Calling	Tools such as DeepVariant use CNNs for high accuracy in detecting genetic variants in short and long reads, aiding fields like medical genetics and evolutionary biology	DeepVariant, DNAscope	Medical genetics, Evolutionary biology
Identification of Pathogenicity	AI models like AlphaMissense and PrimateAI-3D leverage protein structure for variant classification, improving pathogenicity prediction and variant reclassification in diagnostics	AlphaMissense, PrimateAI-3D, RENOVO, StrVCTVRE	Genetic diagnostics, Clinical variant interpretation
Splicing and Copy Number Variation	Non-coding variants such as splicing and CNVs are detected by tools like SpliceAI, which aid in genetic disorder diagnostics and personalized medicine by analyzing functional consequences	SpliceAI, DL-CNV, SVcnn	Genetic disorder diagnostics, Disease mechanism understanding
MHC-Peptide Binding Predictions	AI models like NetMHCpan-4.0 and BigMHC enhance predictions of peptide-MHC binding, crucial for immunotherapy and vaccine development, by using neural networks for improved accuracy	NetMHCpan-4.0, BigMHC, MHCflurry-2.0	Immunotherapy, Vaccine development

### Variant calling

Over the past two decades, Illumina sequencing technologies have dominated variant calling studies with their production of short reads. Numerous tools have been developed specifically for analyzing these short reads, including the Genome Analysis Toolkit (GATK) pipeline. GATK integrates a series of best practice workflows that begin with initial data processing, including quality control and alignment, and continue through variant calling, filtering, and annotation. This comprehensive approach ensures accurate and reliable analysis of genomic data.

Utilizing deep learning techniques, e.g. DeepVariant, excel in accurately identifying genetic variants from next-generation sequencing data. Unlike traditional methods, DeepVariant interprets sequencing data akin to images through convolutional neural networks (CNNs), allowing it to detect subtle genetic variations with exceptional precision. This tool is pivotal in enhancing variant calling accuracy for both single nucleotide polymorphisms (SNPs) and insertions/deletions (indels), thus significantly impacting fields such as medical genetics, personalized medicine, and evolutionary biology. It have showed outperform with other statistical based methods in the short reads [27].

Pacific Biosciences (PacBio) introduced a single-molecule real-time (SMRT) sequencing platform that can generate high-fidelity (HiFi) long reads with an average length of 13.5 kilobases (kb) using a Circular Consensus Sequence (CCS) approach. Oxford Nanopore Technologies (ONT) has changed the sequencing paradigm by introducing sequencers that are portable with real-time data delivery and are able to generate ultra-long reads. Again, DeepVariant is available for those long read variant calling [28, 29]. Also other AI-based variant callers such as DNAscope, which combines GATK’s HaplotypeCaller with a machine-learned genotyping model was developed for short reads and long reads [30].

### Identification of pathogenicity

Identifying a pathogenic variant is a critical process in genetic diagnostics and involves a systematic approach combining multiple sources of evidence. The American College of Medical Genetics and Genomics (ACMG) provides a set of guidelines for variant classification, which includes evaluating population data, computational predictions, functional studies, segregation analysis, and de novo occurrences. Additionally, databases like ClinVar are used to compare findings with existing classifications and supporting evidence [31, 32]. Still lots of variants are classified as a variant of uncertain significance (VUS) when there is insufficient scientific evidence to determine whether the genetic change is benign (harmless) or pathogenic (disease-causing). This uncertainty can arise due to factors such as the rarity of the variant and limited research studies.

AI models like AlphaMissense and PrimateAI-3D have demonstrated remarkable capabilities in predicting the pathogenicity of genetic variants based on structure and function of proteins and evolutionary conservation [33, 34]. AlphaMissense is a deep learning model that leverages protein structural information and evolutionary constraints from related sequences to accurately classify genetic variants as benign or pathogenic [33]. Similarly, PrimateAI-3D is a semi-supervised 3D convolutional neural network that operates on protein structures, enabling it to distinguish between benign and disease-causing variants [34]. SIGMA, also a deep learning model based on protein structural information have been proposed [35]. RENOVO and StrVCTVRE are machine learning tools that reclassify VUS [36, 37]. RENOVO uses random forests to classify variants as pathogenic or benign based on publicly available data, reclassifying 67% of ClinVar VUSs. StrVCTVRE distinguishes pathogenic from benign structural variants overlapping exons, allowing clinicians to prioritize novel SVs and resolve undiagnosed cases.

Those methods have demonstrated remarkable capabilities in predicting the pathogenicity of variants based on structural, evolutionary, and other informative features, enabling the reclassification of many VUS and aiding in the resolution of undiagnosed cases.

### Splicing and copy number variation

Non-coding variants play a pivotal role in the development and progression of various diseases. Splicing variants, which disrupt the normal process of gene expression, can lead to the production of abnormal or non-functional proteins, contributing to genetic disorders and complex diseases. Copy number variations (CNVs), involving deletions or duplications of large DNA segments, can encompass entire genes or regulatory regions, causing gene dosage imbalances and altered expression levels. These changes can directly contribute to genetic disorders or modulate disease susceptibility and severity in complex conditions like autism, schizophrenia, and cancers. Unraveling the impacts of non-coding variants is crucial for understanding disease mechanisms, improving diagnostics, and developing targeted therapies in the era of personalized medicine. SpliceAI, a ML-based tool developed at the Broad Institute, is instrumental in this endeavor [38]. It predicts the effects of genetic variants on splicing, the process of removing non-coding regions from pre-messenger RNA. By analyzing genomic sequences and splicing data, SpliceAI identifies variants likely to disrupt or create new splice sites, leading to aberrant splicing and potential protein dysfunction. Considering genomic context, splice site sequences, and regulatory elements, it provides scores and predictions on the variant’s impact on exon skipping, intron retention, or new splice site creation. This computational approach aids in interpreting non-coding and splice site variants, unveiling their functional consequences and potential involvement in genetic disorders and complex diseases, ultimately contributing to targeted therapies and personalized medicine strategies.

Several novel computational methods for detecting CNVs and structural variations (SVs) from next-generation sequencing data were developed. These include ifCNV, which uses isolation forests for CNV detection without a reference dataset [39]; DL-CNV, an alignment-free deep learning approach for targeted CNV detection [40]; and SVcnn, an accurate deep learning method that improves multi-allelic SV detection from long-read sequencing data [41]. With improved detection of genetic variants like CNVs and SVs, these computational methods facilitate genetic diagnosis, personalized medicine approaches, and understanding disease mechanisms. They overcome limitations of traditional methods, enabling reliable large-scale genomic studies to uncover genetic contributors to diseases and advance precision medicine strategies.

### Predictions MHC-peptide binding

The accurate prediction of peptide binding to MHC molecules is crucial for understanding the immune response and developing effective vaccines and immunotherapies. Traditional methods for MHC-peptide binding prediction, such as position-specific scoring matrices (PSSMs) and quantitative structure–activity relationship (QSAR) models, have limitations in capturing complex patterns and nonlinear interactions [42, 43]. Deep learning techniques, particularly neural networks, have emerged as powerful tools for addressing these challenges and improving the accuracy of MHC-peptide binding predictions. For example, NetMHCpan-4.0 is a widely used computational tool for predicting binding of peptides to any MHC class I molecule based on artificial neural networks [44]. BigMHC comprises an ensemble of seven deep neural networks trained on mass spectrometry data of peptides presented by MHC-I molecules. It also performed transfer learning on data from assays of antigen-specific immune response to further improve the prediction [45]. Other deep learning methods include MHCflurry-2.0 (an ensemble of neural networks) [46], MHCnuggets (many allele-specific LSTM networks for variable length peptides) [47], and MixMHCpred (using positional weight matrices to extract epitope motifs) [48]. The emergence of these advanced machine learning techniques has allowed for significant accuracy gains in MHC-peptide binding prediction.

Artificial intelligence has catalyzed a revolution in genetics through the implementation of machine learning and deep learning techniques, enabling highly accurate variant calling, pathogenicity prediction, splicing analysis, and MHC-peptide binding predictions. The applications of these novel AI methodologies have extended beyond these domains, encompassing the predictions of T cell receptor (TCR) and B cell receptor (BCR)-epitope interactions [49, 50], outperforming human experts in answering genetics-related questions through large language models [51], and facilitating tumor risk prediction and interpretation via multi-omics data integration [52]. Future research directions in this field include the integration of multi-modal data, the development of interpretable models, the implementation of privacy-preserving AI algorithms, and the exploration of generative models. The contributions of AI in genetics span a wide range of applications, including personalized medicine, drug discovery and development, disease prevention, evolutionary biology, and genome editing, driving the advancement of precision healthcare and enhancing our comprehension of the intricate relationships between genotypes and phenotypes.

### Medical utilization

Recent advancements in AI for genetics have profound implications for biomedicine, particularly in precision healthcare. The identification of genetic variants, which are instrumental in understanding disease susceptibility and patient-specific treatment response, has been enhanced through AI-driven techniques. By accurately identifying pathogenic variants, tools such as DeepVariant, AlphaMissense, and PrimateAI-3D enable clinicians and researchers to predict the functional impact of genetic mutations, facilitating targeted treatment approaches for genetic disorders. Similarly, AI’s role in variant reclassification contributes to resolving VUS, allowing for clearer diagnostic interpretations that can impact clinical decision-making. In addition, AI-based splicing and CNV analysis models, including SpliceAI and DL-CNV, support the identification of structural and non-coding mutations, which are key to diagnosing complex diseases like cancer and neurodegenerative disorders. Furthermore, advances in MHC-peptide binding prediction, vital for immunotherapy and vaccine development, allow for a more nuanced understanding of immune responses. By integrating biological and clinical data, AI aids in uncovering novel therapeutic targets and improving the accuracy of disease models, ultimately propelling a shift towards personalized medicine that addresses unique patient genetic profiles.

### AI-enabled immunology data analysis

AI has emerged as a pivotal tool in analyzing immunological data, significantly enhancing our understanding of complex immune responses. The use of AI, particularly in the processing and interpretation of high-dimensional data such as that obtained from flow cytometry and CyTOF (Cytometry by Time-Of-Flight), allows researchers to decipher intricate immune cell interactions and functionalities at an unprecedented scale and depth [53, 54]. ML significantly enhances the analysis of immunological data through various advanced techniques.

### Dimensionality reduction

ML utilizes dimensionality reduction techniques, including Principle Component Analysis (PCA), t-Distributed Stochastic Neighbor Embedding (tSNE), UMAP to simplify the complex datasets generated by flow cytometry and CyTOF [55–57]. By reducing the number of variables while retaining essential information, ML algorithms can more effectively identify meaningful patterns within the data, facilitating a better understanding of immune cell behavior and interactions [53].

### Cell population identification

ML methods facilitate in identifying distinct cell populations within high-dimensional immunological data based on their phenotypic and functional characteristics, allowing for precise mapping of immune cell heterogeneity [53]. Unsupervised and supervised machine learning methods are pivotal for cell-type identification in immunological studies. Unsupervised learning, such as K-means clustering and hierarchical clustering, groups cells based on their inherent similarities in multi-dimensional data sets without prior labels, revealing novel cell populations and states [58]. In contrast, supervised learning methods, including linear discriminant analysis (LDA) classifier and other algorithms of DGCyTOF and DeepCyTOFusing neural network models for cell annotation, rely on labeled training data to classify cells into predefined types, improving the accuracy and robustness of cell-type identification [59]. Integrating both approaches enable a comprehensive understanding of cellular heterogeneity and dynamics, crucial for advancing immunological research and therapeutic development.

### Sample classification

Sample classification in immunological research often leverages cell subset information and predictive modeling using single-cell data to enhance the precision of diagnostics and treatment strategies [53]. By utilizing cell subset information, ML-driven methods, including CITRUS and FloReMi, can categorize samples based on distinct immunological profiles, facilitating a deeper understanding of immune responses across different conditions, such as immune responses to cancer immunotherapy [60]. Predictive modeling of CellCNN and Deep CNN, using single-cell data, allows for the identification of unique cellular signatures that correlate with specific disease states or therapeutic responses [53, 61]. This approach improves the predictive accuracy for patient outcomes, enabling the development of personalized treatment plans that are tailored to individual immunological profiles.

### AI for immunofluorescence image analysis

ML has significantly advanced the analysis of immunofluorescence images, offering enhanced accuracy and efficiency in identifying and classifying cellular patterns.

Our group developed an AI-based models to recognize competent-level antinuclear antibody (ANA) patterns and mixed patterns on human epithelial (HEp-2) cell images according to the International Consensus on ANA Patterns (ICAP) standards [62]. A large dataset of 51,694 HEp-2 cell images with patterns assigned by experienced technologists was used to train six deep convolutional neural network (CNN) architectures. The InceptionResNetV2 model achieved the highest F1 score of 0.86 and kappa of 0.82 on a testing set, demonstrating excellent agreement with experienced human readers for recognizing 11 ICAP competent-level patterns as well as mixed patterns containing up to four overlapping patterns. This highlights the potential of applying transfer learning and fine-tuning publicly available pre-trained CNN models on large clinically-relevant datasets to develop robust automated systems for immunofluorescence image analysis and ANA pattern recognition.

### Cytokine, chemokine and protein analysis

AI’s applications in immunology extend to identifying immune system cell patterns, biomarkers, and therapeutic targets. For instance, ML models are increasingly used to analyze cytokine, chemokine profiles and protein microarray data, which are critical mediators of immunotherapeutic outcome [63]. Our group has demonstrated how ML algorithms can identify patterns and correlations within protein expression profiles that may not be evident through traditional analysis methods [64]. These algorithms, including support vector machines and random forests, can classify proteins based on their expression levels, predict potential biomarkers, and facilitate the understanding of immunogenicity for anti-tumor necrosis factor (TNF)-αbiological therapy in patients with rheumatoid arthritis. By integrating AI with cytokine and chemokine data, researchers can now predict how different individuals’ immune systems might react to specific diseases or treatments, facilitating the development of targeted therapies.

Overall, AI-driven analysis not only enhances the discovery of new biomarkers and therapeutic targets but also improves the accuracy and efficiency of immunological research, paving the way for advanced personalized treatments in immunology.

## Integration of AI in precision medicine

### Synergistic approach to integrating AI analysis of health records, genetics, and immunology data

The integration of AI into precision medicine is not just about leveraging AI in isolated clinical applications but about creating a synergistic approach that encompasses health records, genetics, and immunology data. By applying AI algorithms across these diverse data sets, clinicians can gain a holistic view of a patient’s health status and potential risks. ML models are proving invaluable in identifying high-risk patients in need of rheumatology testing by analyzing EHRs. According to the study published in Nature Communications by Forrest et al., an ML model was developed to predict the necessity for autoimmune disease testing [65]. The model demonstrated high accuracy in identifying patients who should undergo rheumatological evaluation by analyzing longitudinal EHR data from over 161,584 individuals. This approach allowed for earlier detection of the need for autoantibody testing and rheumatology encounters, identifying at-risk patients up to 5 years before traditional clinical assessments would typically do so, thereby accelerating diagnosis and treatment of autoimmune conditions.

Moreover, ML models can significantly enhance the diagnosis of systemic lupus erythematosus (SLE) by integrating EHRs, genomic data, and immunofluorescence image datasets. From our study published in BioData Mining, ML approaches such as Random Forest and Extreme Gradient Boosting were utilized to analyze data from patients with positive ANA [66]. These models effectively identified SLE patients by evaluating clinical features, genomic variations, and specific patterns in immunofluorescence images. Furthermore, the study identified genetic variations associated with lupus in patients with high and low titer ANA, providing deeper mechanistic insights into the disease. The integration of these diverse data sources enables more accurate and timely diagnosis of SLE, facilitating better patient management and treatment outcomes. This AI-supported approach enables the development of highly personalized treatment plans that are predictive, preventive, personalized, and participatory (P4 medicine). Such multi-modal integration not only emphasize the diagnostic process but also enhances the accuracy of treatment outcomes, making it a cornerstone of modern healthcare strategies [67].

### Examples of AI-driven precision medicine platforms and tools for clinical decision support in rheumatology

Several AI-driven platforms and tools have been developed to assist healthcare providers in clinical decision-making [68, 69]. One notable example is IBM Watson Health, which analyzes the meaning and context of structured and unstructured data in clinical notes and reports to provide personalized treatment recommendations [70]. Another is Google’s DeepMind Health, which has developed AI systems for analyzing medical images and electronic health records to support clinical decisions [71].

In the field of rheumatology, AI is being applied to enable more precise diagnosis and treatment selection for patients with rheumatic diseases. One example is an AI-driven precision medicine platform designed to quantify radiographic damage and optimize therapeutic strategies for RA patients [72]. It has demonstrated significant improvements in identifying effective treatments for individual patients, thereby enhancing personalized care. Researchers at Stanford have developed an AI platform called REVAMP that integrates multi-omics data like genomics and proteomics to stratify patients with autoimmune diseases and match them to appropriate therapies [73]. Guan et al. discovered that ML algorithms could predict disease progression and response to therapy in RA patients taking anti-TNF-α inhibitors based on clinical and genomic data. By using AI to analyze complex biomedical information, these platforms clearly facilitate precision rheumatology and optimize care for patients based on their specific disease characteristics and molecular profiles.

### Evaluation of real-world outcomes and clinical benefits of AI-driven precision medicine approaches

Evaluating the real-world outcomes and clinical benefits of AI-driven precision medicine approaches is crucial for their successful implementation in rheumatology practice. A study by Lezcano-Valverde et al. demonstrated that an ML model based on Random Survival Forests (RSF) could accurately predict the risk of mortality in patients with RA using clinical and serological data [74]. Another study by Myasoedova et al. showed that a supervised ML method could predict the response to methotrexate in patients with RA based on clinical and pharmacogenomic data, enabling clinicians to make informed decisions about treatment initiation and optimization [75]. These studies provide evidence for the clinical benefits and real-world impact of AI-driven precision medicine approaches in rheumatology with potential to revolutionize future patient care and outcomes.

## Challenges and considerations

During the implementation of AI based on real world EHRs, several key challenges were encountered. Firstly, EHR data is often messy, inconsistent, and incomplete, making it crucial to ensure high-quality, standardized data for AI training. This requires significant preprocessing and cleaning efforts. For examples, the preprocessing steps to extract simple Blood Pressure (BP) values from clinical notes, structured fields, or imaging reports include standardizing all values to a consistent format like “120/80 mmHg” with uniform units. Outlier detection identifies extreme values (e.g., BP > 200/120 mmHg) for review, while missing BP readings are addressed by either imputing from patient history or neighboring values when appropriate. Quality assurance involves validating BP values against accepted ranges (e.g., systolic < 140 mmHg, diastolic < 90 mmHg), checking for temporal consistency (e.g., sudden BP spikes or drops), and detecting anomalies (e.g., negative BP values). Once cleaned and standardized, BP data can then be used for AI model training, enabling ML models to learn patterns, predict hypertension risk, and monitor patient health. This preprocessing approach applies to various EHR data elements like labs, medications, and diagnoses, ensuring reliable AI training for better predictability and better patient outcomes.

Rigorous testing and validation are necessary to ensure AI algorithms perform accurately and safely in clinical settings, involving extensive training on diverse datasets and continuous monitoring for refinement. Best practices include using a diverse and representative dataset that mirrors real-world scenarios and includes varied demographics, contexts, and edge cases (e.g. under represented population). Continuous monitoring is necessary to track the AI model’s performance post-deployment, detecting any drift or degradation in accuracy over time. Additionally, bias detection and mitigation are essential; analyzing data for biases such as racial, gender, or socioeconomic disparities and implementing strategies to minimize their impact on model predictions is critical. Robust testing should simulate diverse scenarios and edge cases to verify that the model performs well across different situations. These practices help maintain high performance and reliability as well as trustworthy in AI algorithms [76, 77].

Secondly, balancing AI insights with patient privacy is essential. Protecting sensitive patient information while leveraging data for AI necessitates strict adherence to data security protocols and ethical guidelines, ensuring patient trust. Ensuring compliance with privacy regulations is vital for organizations. Best practices include staying informed by continuously monitoring changes in laws and regulations and educating your team about privacy requirements. Hiring data privacy professionals who understand regulations can effectively guide compliance efforts. Implementing controls such as regularly reviewing policies and procedures to ensure alignment with laws and being transparent about data handling practices is essential. Investing in robust security systems to protect sensitive information and limiting access to authorized personnel enhance data security. Establishing a process for reporting non-compliance and defining clear escalation paths for addressing violations ensure prompt and effective responses to any breaches. These practices collectively help maintain compliance with privacy regulations [78].

Thirdly, convincing healthcare professionals to trust and adopt AI recommendations is challenging. Developing user-friendly interfaces and clearly demonstrating the benefits of AI, such as improved diagnostic accuracy and workflow efficiency, are crucial for gaining clinician acceptance and integration into their daily practice. Addressing these challenges is essential for the successful implementation and utilization of AI in EHR systems, ultimately enhancing healthcare delivery and patient outcomes [79].

Finally, while AI holds great promise in clinical decision support, it’s essential to address potential risks and ethical concerns. Recent studies have highlighted several critical considerations [80, 81]: (1) The risk of AI systems perpetuating or amplifying existing healthcare disparities if trained on biased datasets, (2) The potential for over-reliance on AI recommendations leading to decreased clinical autonomy and judgment, (3) The challenge of maintaining transparency in AI decision-making processes, particularly with complex ‘black box’ algorithms, and (4) The need for clear accountability frameworks when AI systems contribute to clinical decisions. These concerns necessitate careful implementation strategies that prioritize patient safety, maintain physician autonomy, and ensure equitable care delivery. Healthcare organizations must establish clear protocols for AI system validation, regular performance monitoring, and mechanisms to detect and address potential biases or errors in AI recommendations. Additionally, clinicians should be trained to appropriately interpret and critically evaluate AI-generated suggestions within the context of their clinical expertise and patient-specific factors.

## Conclusion

In conclusion, this review highlights the transformative potential of AI-driven precision medicine, integrating health records, genetics, and immunology data. By analyzing vast and complex datasets, AI enables unprecedented insights into disease mechanisms and patient-specific responses, facilitating accurate diagnoses and personalized treatments. To fully utilize AI’s potential in revolutionizing healthcare, continued research, development, and clinical integration are crucial. Investing in and adopting AI-driven precision medicine strategies will lead to a new era of personalized patient care and medical breakthroughs, ultimately improving outcomes and driving innovation in rheumatology.

## Data Availability

The datasets during and/or analysed during the current study available from the corresponding author on reasonable request.
